# The role of exercise in modulating NAD+ metabolic enzymes: mechanisms and health benefits

**DOI:** 10.3389/fspor.2026.1863337

**Published:** 2026-09-07

**Authors:** Anas, Habiba Sundus, Sohrab Ahmed Khan, Syed Suhaib Ahmed, Punitha Muruganantham, Inamul Hasan Madar

**Affiliations:** 1 Department of Occupational Therapy, Yenepoya Faculty of Allied and Healthcare Professions, Yenepoya (Deemed to be University), Mangalore, Karnataka, India; 2 Department of Physiotherapy, Jamia Hamdard, New Delhi, India; 3 Department of Pharmaceutics, Yenepoya Pharmacy College & Research Centre, Yenepoya (Deemed to be University), Mangalore, Karnataka, India; 4 Centre for Integrative Omics Data Science (CIODS), Yenepoya (Deemed to be University), Mangalore, Karnataka, India

**Keywords:** exercise physiology, mitochondrial function, NAD+ metabolism, nicotinamide phosphoribosyltransferase (NAMPT), sirtuins

## Abstract

Nicotinamide adenine dinucleotide (NAD+) is a critical coenzyme in cellular metabolism, acting as a substrate for NAD+-dependent enzymes such as sirtuins, poly(ADP-ribose) polymerases (PARPs), and CD38, which are involved in essential cellular processes including DNA repair, gene expression, and mitochondrial function. Exercise has been identified as a potent modulator of NAD+ metabolism and also influencing the expression and activity of enzymes involved in NAD+ biosynthesis and consumption. Our review focuses on the molecular mechanisms through which exercise regulates NAD+ metabolic enzymes, more in relation to aerobic and resistance exercise. Evidence indicates that exercise enhances the activity of key enzymes, such as nicotinamide phosphoribosyltransferase (NAMPT) and sirtuins, thereby improving mitochondrial efficiency, oxidative metabolism, and cellular homeostasis. Additionally, exercise has also been shown to mitigate the age-related decline in NAD+ levels, offering a potential strategy for addressing metabolic dysfunctions and age-associated diseases. Despite these findings, the exact molecular pathways underlying the exercise-induced modulation of NAD+ metabolism remain unclear and require further investigation.

## 1 Introduction

One of the essential coenzymes in cellular metabolism, nicotinamide adenine dinucleotide (NAD+), serves as a substrate for NAD+-dependent enzymes, including sirtuins, poly(ADP-ribose) polymerases (PARPs), and CD38, which is also a vital redox carrier in oxidative phosphorylation and glycolysis (1). These enzymes play a critical role in preserving cellular homeostasis by regulating key cellular functions, including gene expression, DNA repair, mitochondrial function, and inflammation (2). Understanding the variables that affect NAD+ bioavailability and enzymatic activity is crucial since dysregulation of NAD+ metabolism has been linked to ageing, metabolic disorders and neurological illnesses due to its important role (3).

Exercise is well known for its powerful regulation of cellular and systemic metabolism. The expression and activity of important enzymes involved in the NAD+ biosynthesis and salvage pathway, including as nicotinamide phosphoribosyl-transferase (NAMPT), nicotinamide mononucleotide adenylyl-transferases (NMNATs) and sirtuins, have been shown to be enhanced by exercise-induced metabolic adaptations (4). The resistance to metabolic stress, increased oxidative metabolism and higher mitochondrial efficiency are all facilitated by these molecular changes (5). Significantly, exercise has been proposed as a non-pharmacological intervention that may counteract age-related NAD+ decline, thereby reducing age-related metabolic dysfunction and extending lifespan (6).

The precise molecular processes underlying exercise-induced regulation of NAD+-dependent enzymes remain poorly understood, despite increased interest in the relationship between exercise and NAD+ metabolism. With a focus on the regulation of NAD+ biosynthesis enzymes and their downstream metabolic effects, our review aims to provide a thorough synthesis of the state of the art on the impact of exercise on NAD+ metabolic pathways.

### 1.1 Literature search strategy

A comprehensive literature search was conducted across the PubMed, Scopus, Web of Science, and Google Scholar databases to inform a narrative review. Literature from January 2000 to December 2024 was included. Search terms were “NAD+”, “nicotinamide adenine dinucleotide”, “exercise”, “physical activity”, “NAMPT”, “SIRT1”, “SIRT3”, “PARP”, “CD38”, “mitochondrial function”, “ageing”, “oxidative stress” and “metabolic health” in various combinations. Studies were selected if they were peer-reviewed articles in English that examined the effect of exercise on NAD+ metabolism, NAD+-dependent enzymes, mitochondrial function, ageing, and metabolic health in human or animal models. Reviewing articles was also undertaken to identify other relevant research. Studies not related to an exercise training intervention, conference abstracts, editorials, and those not written in English were excluded. As the research area is vast and relatively new, a narrative review was used to bring together existing evidence on the modulation of NAD+ metabolic pathways by exercise and how this affects health and disease.

## 2 NAD+ biosynthesis pathways

The cellular activities that support different metabolic functions depend on NAD+ (7). NAD+'s traditional function as a co-enzyme in numerous basic metabolic activities, including glycolysis, fatty acid beta oxidation and the tricarboxylic acid cycle, is to catalyze cellular redox reactions and then be condensed to NADH (8). Along with these functions, NAD+ is essential for NAD+-consuming enzymes such as sirtuins, poly-ADP-ribose polymerases (PARPs) and CD38/157 ectoenzymes (9–11). It is known that these NAD+-consuming enzymes mediate a number of essential cellular functions (7). New data also show that NAD+ is a key signaling molecule that plays a role in ageing, DNA repair, inflammation, mitochondria, and cellular resilience (12). The synthesis of NAD+ requires five main precursors and intermediates: nicotinamide, nicotinic acid, nicotinamide riboside (NR), nicotinamide mononucleotide (NMN) and tryptophan. By converting the amino acid tryptophan to nicotinic acid mononucleotide (NaMN) via a series of enzymatic processes, NAD+ may be produced from scratch (13, 14). NAD+ synthetase amidates NaMN to NAD+ after NMN/NaMN adenylyl-transferases (NMNATs) convert it to nicotinic acid dinucleotide (NaAD+).

**Figure 1:**
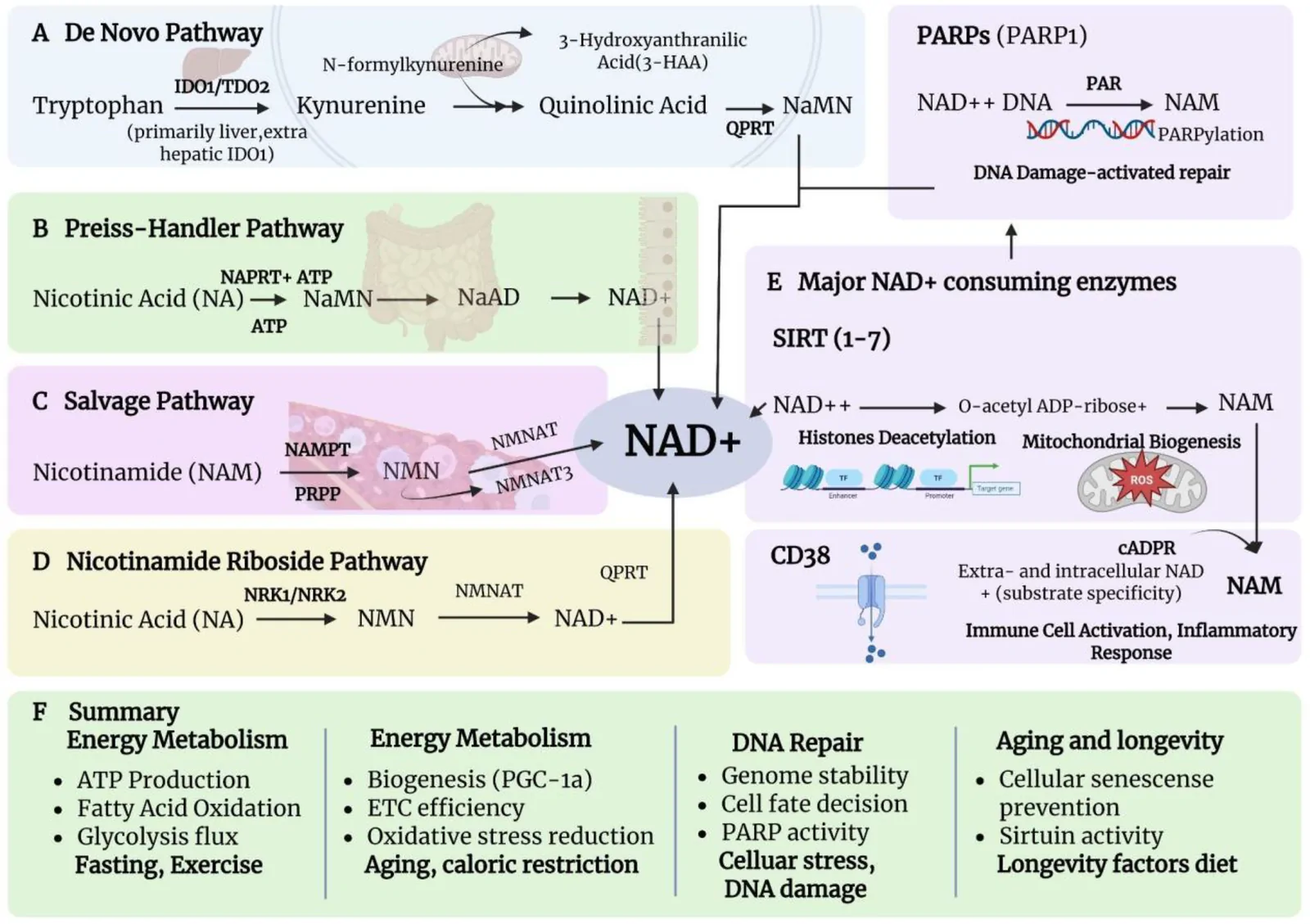
NAD⁺ biosynthesis pathways and major NAD+-consuming enzymes. Schematic overview of nicotinamide adenine dinucleotide (NAD⁺) biosynthesis via the *de novo* pathway from tryptophan, the Preiss–Handler pathway from nicotinic acid and the salvage pathway from nicotinamide and nicotinamide riboside, highlighting NAD⁺ utilization in cellular energy production and DNA repair. Created with BioRender.com. Available at: https://app.biorender.com/illustrations/6a2929c905edd070cec5dea6?slideId=c51249f9-ee0f-424f-abef-eb143c142769.

Figure 1 summarizes the major pathways through which NAD+ is synthesized, the *de novo* pathway, Preiss–Handler pathway, and salvage pathway.

The enzyme, which is rate-limiting in this route, nicotinamide phosphoribosyl-transferase (NAMPT), transforms nicotinamide into NMN, a crucial NAD+ intermediate. NMNATs subsequently convert NMN into NAD+ (15, 16). Cellular NAD+ levels are regulated in large part by NAMPT (13, 14). Nicotinic acid phosphoribosyl-transferase (NPT), on the other hand, transforms nicotinic acid into NaMN (15, 16). NMRK1 and NMRK2 (sometimes called NRK1 and NRK2) are nicotinamide ribose kinases that must phosphorylate NR1 in order to convert it to NMN. Sufficient NAD+ production must be maintained for cells to survive and function. The activity of NAD+-dependent enzymes involved in essential cellular processes is also significantly affected by deviations from normal NAD+ homeostasis, as is the NAD+/NADH redox pool (15).

## 3 Exercise and NAD+ metabolism: molecular pathways

### 3.1 Impact of exercise on NAD+ biosynthesis

The impact of exercise on NAD metabolism differs markedly between acute exercise and chronic exercise training. While acute exercise causes metabolic stresses and signalling that are transient, chronic exercise training will lead to longer-term changes in mitochondrial function, NAD+ biosynthesis, and enzyme activity.

The salvage route is the principal mechanism in mammals that keeps intracellular NAD+ levels stable (17). In particular, this review examines how exercise regulates enzymes in the salvage pathway. Prior investigations have mostly examined how the rate-limiting enzyme NAMPT responds to exercise, however some have also included nicotinamide riboside kinase 1 (NRK1), NMNAT and NRK2.

#### 3.1.1 Exercise-induced changes in NAMPT levels

##### 3.1.1.1 Human studies

For younger people (under 35) and older people (over 55), aerobic exercise training increased NAMPT levels in skeletal muscle tissue by 12% and 28%, respectively. The NAMPT levels of younger and older persons, however, increased by approximately 25% and 30%, respectively, following resistance exercise training. These results imply that the age-related decrease in skeletal muscle NAMPT levels may be reversible with exercise training (18). Resistance training has been shown to increase NAMPT protein levels in the skeletal muscles of people aged 59 ± 4 years. Also, skeletal muscle NAMPT protein expression is doubled in endurance-trained athletes as compared to sedentary diabetics, obese people and nonobese people. Three weeks of exercise training resulted in a 127% rise in NAMPT protein levels in sedentary nonobese participants, which was directly correlated with mitochondrial content. Similar increases in NAMPT expression were reported in skeletal muscles of football players (19, 20).

##### 3.1.1.2 Animal studies

Other investigations have sought to determine whether exercise and NAMPT expression interact. Despite not improving insulin resistance, overexpression of NAMPT in muscle tissue was shown to protect mice against obesity induced by a high-fat diet. Mice whose muscles overexpress NAMPT were able to achieve three times the exercise tolerance of wild-type mice via voluntary exercise training. Furthermore, mitochondrial gene expression increased significantly. These results imply a relationship between increased skeletal muscle NAMPT activity and exercise, leading to enhanced exercise endurance (21, 22). Not all of the investigations showed consistent increases in NAMPT levels. After four weeks of voluntary running wheel training and treadmill, for example, Hokari et al. (23) did not observe any appreciable changes in NAMPT expression in the skeletal muscle of rats.

In recent research on human systemic NAD+ metabolism, it was shown that in those aged 18–35 with better aerobic fitness, acute moderate-intensity cycling exercise caused the release of NAMPT from extracellular vesicles (EVs). For those with better aerobic fitness (ages 50–70) and those with lower aerobic fitness (ages 18–35), this reaction was less significant. The observed reaction was correlated with age and fitness levels; notably, those aged 50–70 with poorer aerobic fitness did not show an increase in NAMPT release. In recipient cells, the release of extracellular vesicles - extracellular nicotinamide phosphoribosyltransferase (EV-eNAMPT) during exercise was shown to increase NAD+ levels, suggesting that exercise may help prevent age-related declines in NAD+ (24). However, not all human studies showed consistent increases in NAMPT levels. For example, the quantity of NAMPT in abdominal subcutaneous fat did not change in either young or elderly individuals after 6 weeks of high-intensity interval training (HIIT) (18). However, in young, healthy people, a 6 month circuit training intervention resulted in a considerable decrease in blood NAMPT levels (25). Furthermore, four weeks of sprint interval training (SIT) had no effect on plasma NAMPT levels (26). The kind of tissue, the type of exercise, the intensity and the length of time spent exercising may all affect the effects.

The different effects of exercise on NAMPT expression might be due to variations in tissue, type of exercise, training duration, age, and metabolic status. The expression of NAMPT tends to be increased in response to metabolic stress in tissues which require high levels of NAD+-mediated energy production such as skeletal muscle, and is less responsive in adipose tissue. It is generally accepted that endurance exercise mainly stimulates AMPK–NAMPT–NAD+ signalling pathway by altering the AMP/ATP ratio, which leads to increased NAD+ biosynthesis and mitochondrial adaptations. The AMPK–NAMPT–NAD+ axis is known to be an important metabolic axis that is involved in cellular energy sensing and mitochondrial adaptation that regulates energy homeostasis in the whole body (2). However, NAMPT could be activated by resistance exercise via muscle remodelling and hypertrophic signalling pathways. There are also age-related differences that might account for this variability, as older adults tend to have lower baseline NAD+ levels and reduced mitochondrial function, allowing for larger relative increases after exercise training. In addition, variations between the studies may be attributed to the intensity, duration, timing of sample collection and the level of the exercise outcome measured (mRNA, protein, or activity).

#### 3.1.2 Effects of exercise on NMNAT, NRK1 and NRK2

Other important enzymes in the salvage process include NRK1 and NRK2. Although there is limited data on the effects of exercise training on these enzymes, most studies have focused on cardiac and skeletal muscle. In Drosophila with variable cardiac dSir2 expression, Deng et al. (27) discovered that two weeks of climbing exercise improved cardiac cycle length and decreased diastolic dysfunction index by increasing the expression of many proteins, including silent information regulator 2 (Sir2). According to the scientists, exercise increased NMNAT expression in the heart, potentially affecting Sir2 regulation through elevated NAD+ levels. Exercise may help prevent cardiac ageing by activating the dSir2/forkhead box o (FOXO)/superoxide dismutase (SOD) and dSir2/FOXO/brummer (bmm) pathways. The NMNAT/NAD+/Sir2 pathway may represent a defence mechanism against lipotoxic cardiomyopathy in Drosophila, according to further study by the same group that found increased cardiac NMNAT expression and NAD+ levels during exercise (28). However, a 6-week high-intensity interval training (HIIT) program did not substantially change the abundance of NMNAT1/2 in subcutaneous fat and both aerobic and strength training had modest effects on the levels of NMNAT1 in human skeletal muscle (18).

While NRK2 is exclusive to skeletal muscle and helps maintain NAD+ levels and regulate muscle function, NRK1 is present in a variety of tissues. NAD+ availability is increased by both enzymes’ comparable responses to NR and NMN (29). Research has not shown that exercise affects NRK1/2 levels. NRK1/2 levels in human skeletal muscle and abdominal subcutaneous adipose tissue appear to be unaffected by age or by different types of exercise, such as aerobic, resistance, and high-intensity interval training (HIIT) (18).

### 3.2 Exercise-induced regulation of NAD+ consuming enzymes

#### 3.2.1 Sirtuins and exercise adaptations

ADP ribosyl-transferase activity (SIRT4 and SIRT6) and NAD+-dependent deacetylase activity (SIRT1-3, SIRT5-7) are both shown by the mammalian sirtuin family, which comprises SIRT1–7. Their functions in preserving cellular balance, such as improving energy metabolism, DNA repair, preventing oxidative stress, guaranteeing genome integrity and other biological processes are determined by differences in their locations within cells and the substrates they choose (30–33). The way sirtuins, especially the deacetylases SIRT1 and SIRT3, respond to physical exertion is similar to that of NAD+ (34). The effects of exercise on these enzymes, which are among the most studied NAD+-utilising enzymes, have been the subject of several investigations.

##### 3.2.1.1 Human studies

Single exercise sessions have been shown to have no discernible effect on SIRT1 or SIRT3 mRNA levels in skeletal muscle in studies with older participants (35). Acute or solitary exercise may increase SIRT1 mRNA expression in skeletal muscle in young people who do not exercise regularly, according to the majority of studies (35, 36). Participants with long-standing exercise routines, however, did not show this impact (35, 37). The present investigation indicates that acute or single-session exercise either decreases or has no discernible effect on SIRT3 mRNA levels in young participants’ skeletal muscle (35, 37). Whether or not the individuals have developed exercise routines and the particular exercise technique used have an impact on these results. Young, recreationally active people's skeletal muscle's SIRT1 protein expression is affected by different exercise intensities after two weeks of brief high-intensity interval training (38, 39). In skeletal muscle of overweight, inactive people, interval training for 3 weeks rather than 2 weeks has been shown to alter SIRT1 expression (40, 41). In the skeletal muscle of young people who exercise regularly, a 6-week High-intensity interval training (HIIT) program dramatically raised SIRT1 activity while decreasing SIRT1 protein levels (42). In middle-aged or older persons, some research has shown no discernible effect of 10 or 12 weeks of exercise training on SIRT1 expression, whereas other studies demonstrate an increase in SIRT1 activity (43, 44). Furthermore, in the skeletal muscle of untrained, healthy individuals, SIRT1 mRNA expression may be elevated by long-term (64-week) exercise training (45). In young people, exercise training for three to six weeks had a minimal impact on SIRT3 levels. Serum (46) showed an increase in SIRT3 mRNA, whereas skeletal muscle (47, 48) did not (46). Skeletal muscle SIRT3 protein expression increased in young, elderly, and obese individuals during prolonged endurance exercise (49, 50). Skeletal muscle from obese teenagers did not show a substantial change in SIRT3 expression after 12 weeks of resistance training (50). Age, physiological state, type of exercise, and duration all affect the alterations in SIRT1 and SIRT3 expression that exercise induces in humans, according to research.

The differential behavior of SIRT1 and SIRT3 in response to exercise may be attributed to the differential metabolic needs of these tissues, and/or differences in the activation of the upstream signaling pathways. This boost in NAD+ during exercise leads to greater SIRT1 activity, deacetylation of PGC-1α, and mitochondrial biogenesis. SIRT3, which resides mainly in mitochondria, is sensitive to mitochondrial NAD+ pools and can control oxidative metabolism by deacetylation of important mitochondrial enzymes. It is generally believed that endurance-type exercise causes greater AMPK–SIRT1–PGC-1α activation, while high-intensity exercise causes transient metabolic stress, which could have differential effects on sirtuin expression. This is also a possible explanation for older people sometimes not responding as well as younger people, as age-related declines in mitochondrial plasticity and NAD+ levels may be responsible. While human studies provide valuable translational evidence, animal studies offer important mechanistic insights into tissue-specific responses and long-term adaptations induced by exercise.

##### 3.2.1.2 Animal studies

It is unclear how acute or short-term exercise (less than 4 weeks) affects SIRT1 and SIRT3 expression in mouse skeletal muscle and the brain, due to variations in exercise duration and technique (45, 51). Furthermore, six weeks of voluntary exercise had no discernible effect on SIRT1 protein expression in the skeletal muscle of young animals (52), while six weeks of high-intensity interval training caused a reduction in this expression (53). After 12 weeks of swimming exercise, the hippocampal expression of SIRT1 protein changed in opposing ways in young and elderly mice (54). Moreover, it is notable that adult and middle-aged rats’ soleus muscles, but not their gastrocnemius muscles, showed enhanced SIRT1 protein expression after 12 weeks of swimming activity (55). SIRT3 expression was elevated in a variety of tissues in both young and elderly animals after four weeks of exercise training (56). Six weeks of treadmill activity had no discernible effect on SIRT3 protein expression in the brains of young animals, consistent with SIRT1-related findings (57, 58). Nonetheless, six weeks of swimming exercise, but not treadmill activity, increased SIRT3 protein expression in the hippocampus of elderly rats (57). There were conflicting results from longer-term (8–20 week) exercise trials. In older MI model mice, prolonged short-term swimming activity, but not long-term swimming, increased SIRT3 protein levels (59). Following eight weeks of treadmill activity, SIRT3 expression in the cerebral cortex of CD-1 mice declined; however, after sixteen weeks of exercise, it rose (60). It has been shown that 20 weeks of exercise in amyloid precursor protein/presenilin-1 (APP/PS1) transgenic mice increases SIRT3 protein levels in the hippocampus (61), but does not clearly regulate SIRT3 levels in the liver (62). Notably, SIRT3 protein levels in the hearts of young SD rats and the muscles of rats with poor or high running ability were not significantly affected by a year of exercise in many investigations (63, 64). In summary, the available evidence suggests that exercise stimulates the activity of SIRT1 and SIRT3, which leads to increased mitochondrial function, oxidative metabolism, and resistance to cellular stress. The differences between studies may, however, be due to the differences in age, training status, type of tissue analyzed and the type of exercise done. Future studies are needed to standardize exercise interventions and assessment methods so as to better describe adaptations mediated by sirtuins.

**Figure 2:**
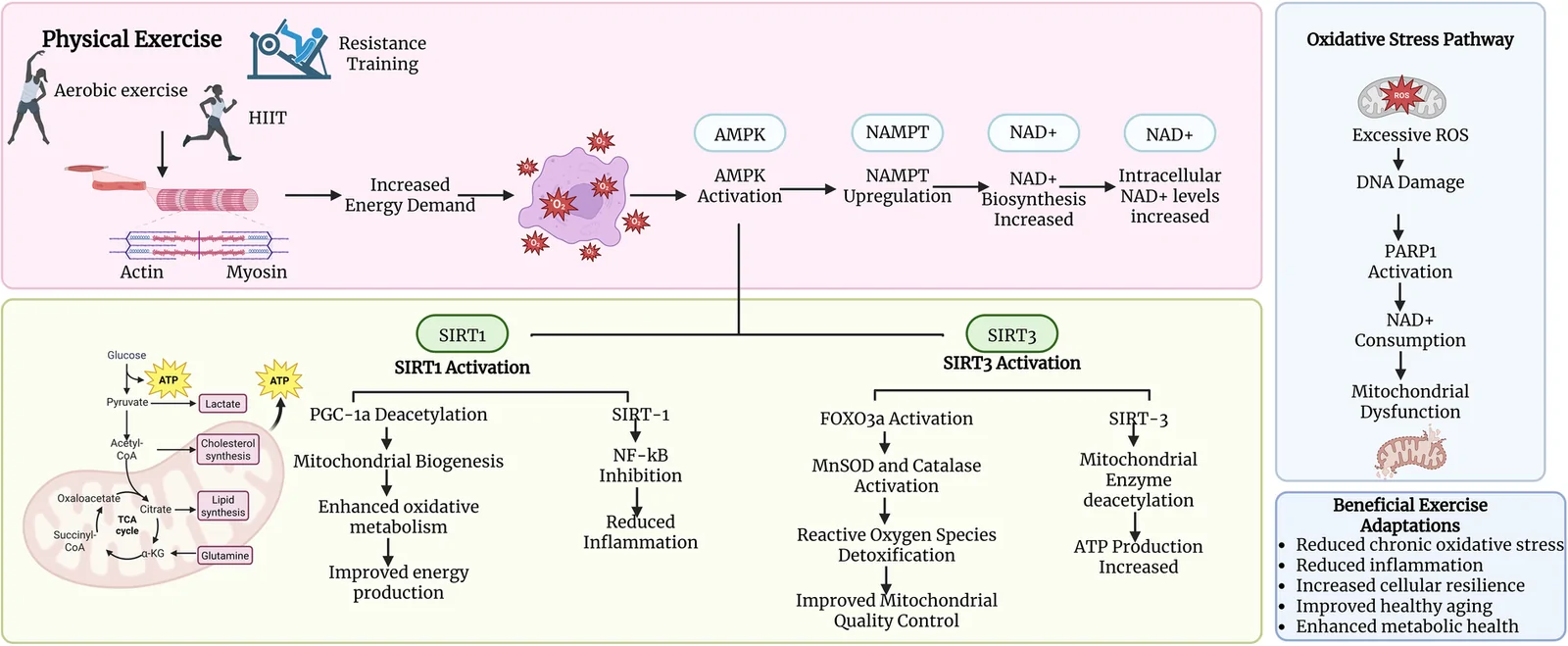
Exercise-induced modulation of NAD⁺ metabolism, sirtuin signaling and mitochondrial adaptation. Physical exercise enhances NAD⁺ biosynthesis and activates key molecular pathways, resulting in improved mitochondrial function, metabolic health and reduced ageing-related decline. Created with BioRender.com. Available at: https://app.biorender.com/illustrations/6a2929c905edd070cec5dea6?slideId=5d35dffb-80d8-46a4-a6bf-0cfbc6fee8f7.

Major molecular pathways by which exercise regulates NAD+ metabolism and mitochondrial function are depicted in Figure 2.

Peroxisome proliferator-activated receptor *γ* coactivator 1 alpha (PGC-1α), nuclear factor kappa-B (NF-κB), Tp53 and FOXO are just a few of the proteins that SIRT1, which is found in both the cytoplasm and the nucleus, interacts with to regulate a variety of physiological processes. SIRT3 is mostly found in the inner mitochondrial membrane, where it is essential for regulating antioxidant defence and energy metabolism (65–67). Since SIRT1 and SIRT3 are important regulators of metabolic adaptation and are modulated by exercise, many researchers have studied how exercise training alone affects the molecular pathways linked to SIRT1 and SIRT3. Exercise prevents kidney cell (68) and cardiomyocyte (69) apoptosis by regulating the SIRT1/p53 pathway, thereby improving early diabetic kidney damage and protecting the heart, according to research on other SIRT1 targets such as Tp53 and NF-κB. The decrease in the inflammatory response in the brain tissue of diabetic (70) and ApoE−/− (71) mice is directly linked to exercise's ability to activate SIRT1 and inhibit the NF-κB signaling pathway. SIRT3 plays a key role in regulating antioxidant responses and metabolic alterations induced by exercise (72). Studies reveal a strong correlation between SIRT3 levels and ATP synthesis during exercise. Skeletal muscle ATP levels were shown to drop significantly in experiments using SIRT3 knockout mice, resulting in decreased muscular endurance during exercise (73). Additionally, Donniacuo et al. (70) found that exercise may reduce oxidative stress in myocardial infarction by increasing catalase and manganese superoxide dismutase (MnSOD) expression and activating the SIRT3/FOXO3a pathway. Exercise has also been shown to improve the hippocampus's SIRT3/MnSOD pathway, which helps to repair the neuronal damage and cognitive impairment brought on by a high-fat diet (HFD) (74).

The sirtuin family is intimately associated with the anti-ageing benefits of exercise. Studies have shown that swimming exercise inhibits inflammatory signalling and mitochondria-dependent apoptotic pathways in the aged rat hippocampal region by activating pathways such as insulin-like growth factor (IGF)/PI3K/AKT and AMPK/SIRT1/PGC-1α (54).

#### 3.2.2 Influence of exercise on PARP activity

Both internal and external environmental variables that impact genomic DNA may cause DNA damage. NAD+ is broken down by PARP into NAM and ADP-ribose, which are subsequently used to poly-ADP ribosylate PARP1 and other receptor proteins to create PARPADP-ribose branched chains that are essential for repairing DNA damage. The very active PARP1 uses up a lot of NAD+ when DNA damage occurs (75). Furthermore, a crucial component of the apoptosis cascade, caspase-3, uses PARP as a cleavage substrate. Caspases-3 cleaves PARP into two pieces, 89kDa and 24kDa, during the early stages of apoptosis, which facilitates the later apoptotic processes (76). Numerous research studies have looked at how exercise affects PARP. Two of the studies by Maria (77) and Cobley (78) et al. show that PARP1 activity or levels are associated with aerobic fitness in young people under acute exercise regimens. For example, after a single High-intensity interval training session, PARP1 levels in the skeletal muscles of young people who participated in long-term endurance training were constant, but PARP1 levels rose in young people who were not trained (78, 79). Age, tissue type, species and exercise intensity may all have an effect on how acute exercise affects cleaved PARP levels. In particular, 24 h after acute moderate-intensity exercise, mouse cardiac tissue showed a substantial rise in cleaved PARP protein levels, which returned to baseline 48 and 72 h later (80, 81). Cleaved PARP1 levels in skeletal muscle of young people tended to rise both immediately and three days after acute High-intensity interval training. On the other hand, older people's levels of cleaved PARP1 did not alter before or after exercise (78, 82).

The effects on cleaved PARP levels are further investigated by regular exercise training programs. Studies show that aerobic exercise or High-intensity interval training (HIIT) reduces the amounts of cleaved PARP in many tissues in a variety of pathological animal models, such as aging (83), menopause (76) doxorubicin-induced heart damage (81, 84) and diabetic cardiomyopathy (85). This implies a decrease in apoptosis inside cells. However, as shown by the elevated expression of PARP and cleaved PARP, respectively, prolonged eccentric activity (86, 87) and excessive endurance exercise (87) cause damage to the heart and skeletal muscle. This implies an increase in apoptosis and DNA damage. Other investigations also found alterations related to sex and tissue. For instance, the cleaved to total PARP1 ratio in the heart muscle of healthy rats decreased after 12 weeks of HIIT (88). While there was no discernible change in slow-twitch muscle tissues, this exercise raised the ratio in fast-twitch muscle tissues (89). Following four weeks of acrobatic training, it was shown that female rats with chronic cerebral hypoperfusion had greater levels of PARP expression in their hippocampal regions than did male rats. This result proposes that exercise might have a sex-specific impact on DNA protection (90, 91).

#### 3.2.3 Role of CD38 in exercise responses

The NAD+-consuming enzyme CD38 has ADP-ribosyl cyclase and glycohydrolase functions. It catalyzes the transformation of NAD (P)+ into vital second messengers that are essential for Ca2+ mobilization, such as cADPR, NAAD(P) and ADPribose. CD38 plays a crucial role in maintaining Ca2+ homeostasis (92), which is necessary for excitation-contraction coupling in cardiomyocytes and skeletal muscle cells. Furthermore, Ca2+ is essential for a number of cellular processes and exercise-related metabolic processes. One research on skeletal muscle cells by Park et al. (93) showed that CD38 plays a crucial role in muscular contraction during exercise. Exercise or β-adrenergic receptor activation may raise CD38 expression by phosphorylating the cyclic AMP response element-binding protein, which results in the synthesis of cADPR. This chemical increases muscle contractility and sarcoplasmic reticulum Ca2+ uptake by acting as a sarcoendoplasmic reticulum Ca2+-ATPase activator. Possibly as a compensatory mechanism to satisfy the increased NAD+ demand during blood glucose level control, CD38 is downregulated in response to glucose pre- and post-SIT (Sprint interval training), according to one research on the effects of 4 weeks of SIT on the sirtuin/NAD+ system (60). According to recent research, amyloid precursor protein/presenilin-1 (APP/PS1) mice's hippocampal overexpression of CD38 may be inhibited by 12 weeks of treadmill activity. This lowers NAD+ consumption and improves NAD+ utilization by SIRT1, another consuming enzyme. In the end, this procedure improves mitochondrial dysfunction in the hippocampus of Alzheimer's disease mice by upregulating the NAD+/SIRT1/FOXO1/3a signaling pathway (94, 95). Despite these results, nothing is known about how exercise affects CD38, which calls for further study to determine how different forms of exercise could affect CD38 levels in different tissues.

### 3.3 Oxidative stress, reactive oxygen species and NAD+ metabolism

Exercise has been shown to lead to the generation of reactive oxygen species (ROS) and in particular from mitochondrial activity of the electron transport chain. High levels of ROS can cause harm to cellular macromolecules, but moderate increases of ROS during exercise have an important role as signaling molecules, and can produce adaptive responses (hormesis). During exercise, ROS activate the AMPK and PGC-1α signaling pathways, leading to increased mitochondrial biogenesis and antioxidant defenses.

There is a strong relationship between oxidative stress and NAD+ metabolism. High ROS levels can cause damage to DNA, which in turn can activate poly (ADP-ribose) polymerase-1 (PARP1), a large NAD+-using enzyme. Excessive activation of PARP can lead to rampant loss of NAD+ which can make less NAD+ available for cellular processes mediated by sirtuins. Accordingly, chronic oxidative stress could affect mitochondrial activity by reducing SIRT1 and SIRT3 activity.

SIRT1 and SIRT3 are factors involved in redox homeostasis. SIRT1 inhibits inflammatory signaling by inhibiting NF-κB, which in turn, limits ROS production and cellular inflammation. In parallel, SIRT3 promotes mitochondrial antioxidant protection by deacetylating and activating MnSOD and FOXO3a, which promotes ROS detoxification. These pathways are activated during exercise and contribute to better mitochondrial function, more efficient oxidative phosphorylation and protection against metabolic dysfunction with age.

## 4 Exercise, aging and NAD+ metabolism

### 4.1 Age-related decline in NAD+ levels

In humans, NAD+ concentration has been observed to decrease in skin samples with growing age. Although there is no precise calculation of decline, the average concentration which appeared to decrease at least was about 50% over the course of adult aging and several fold-lower in adults when compared to newborns (96). This age-related changes in NAD+ metabolism are corroborated by metabolomic analyses of circulating metabolites showing significant age related changes in metabolites associated with energy metabolism and redox homeostasis (12). NAD(H) was also reported to decline by close to 14% in the cerebrospinal fluid of subjects with more than 45 years of age as compared to those with less than or equal to 45 years (97). Two studies which are MRI-based, have also provided evidence for NAD+ decline in human brains with growing age, ranging from ∼10% to ∼25% between young adulthood and old age (98, 99). In contest, a different study that has been made available prior to peer review suggests that there is not a consistent difference observed in NAD+ content between the brains of young and old humans (100, 101). Therefore, larger studies may still be required to provide a definitive answer as to how the NAD+ content of human brain changes with age. In a small cohort of older individuals NAD+ content was also seen to decrease in monocyte-derived macrophages.

**Figure 3:**
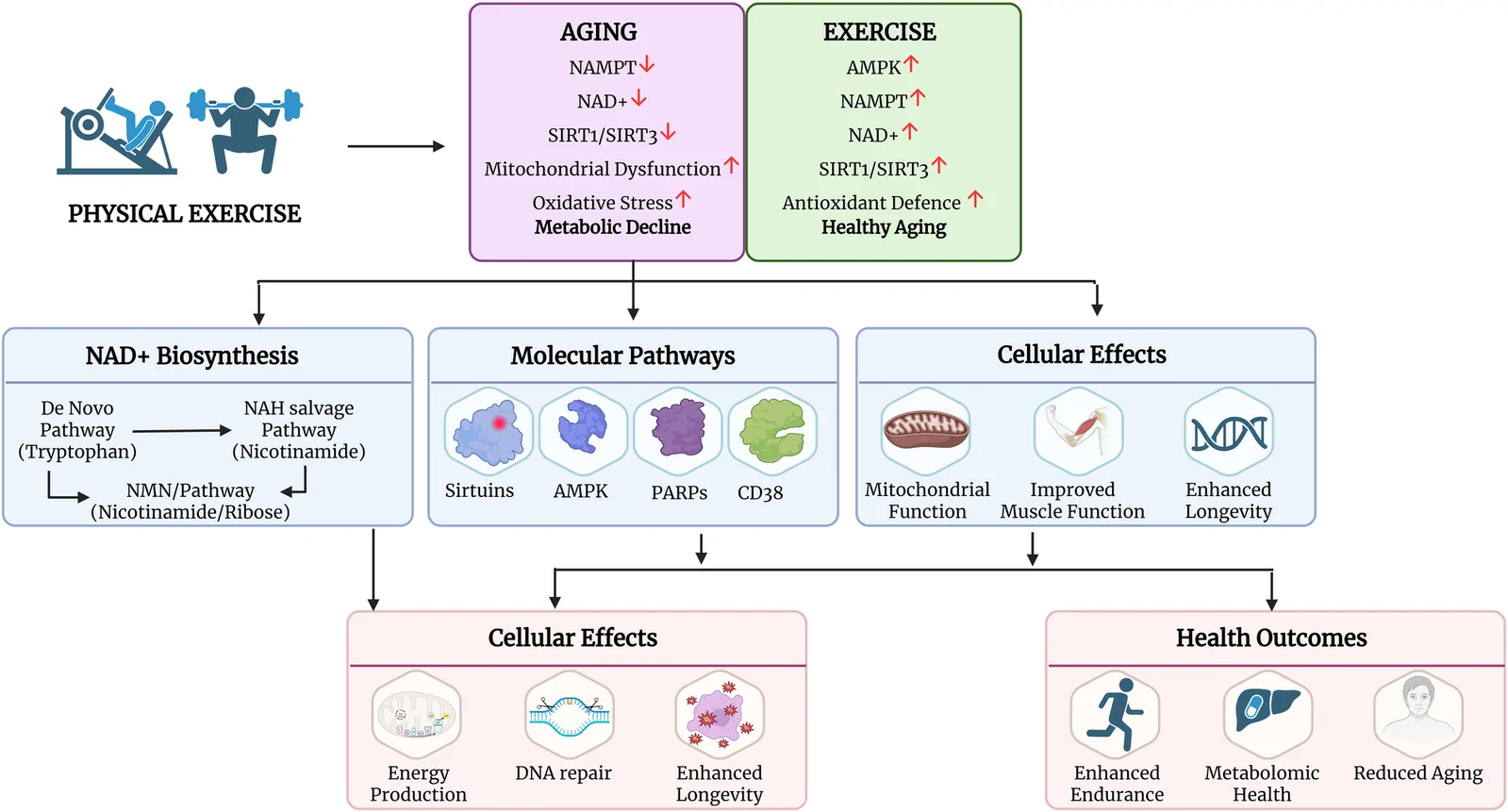
Relationship between exercise, aging and NAD+ metabolism. Lifting weights stimulates AMPK signaling which increases NAMPT expression and thus increases NAD+ biosynthesis. NAD+ stimulates SIRT1 and SIRT3 to regulate PGC-1alpha, FOXO3a and antioxidant defense pathways. These adaptations increase mitochondrial biogenesis, oxidative metabolism, resistance to cellular stress, and metabolic health and reduce age-related decreases in mitochondrial function and NAD+. Created with BioRender.com. Available at: https://app.biorender.com/illustrations/6a2929c905edd070cec5dea6?slideId=c15095f2-49d7-4d0c-8119-70e6e5fd6f8a.

The relationship between exercise, ageing and NAD+ metabolism is summarized in Figure 3.

Finally, plasma NAD+ levels (which are at nanomolar concentrations, 3–4 orders of magnitude below tissue concentrations and have an uncertain significance) were recently shown to decline steeply with age in humans in a study that used improved methodology as compared to a prior report that showed only a modest trend (102–104). Therefore, a decline in NAD+ concentration in at least few tissues appears to be a conserved feature of aging across species which also include humans. This reduction in NAD+ has been linked to aging and is thought to be a key indicator of senescence and a driver of mitochondrial dysfunction, genomic instability, and cellular stress responses (12).

### 4.2 Exercise as an anti-aging strategy

Physical exercise exerts profound regulatory effects on aging-related metabolic dysfunction by modulating key enzymatic pathways involved in NAD+ metabolism, mitochondrial biogenesis and oxidative stress management. Activation of SIRT1 and SIRT3 through exercise has been shown to enhance glucose metabolism, thereby delaying the onset of age-related sarcopenia and diabetes-associated metabolic decline. Furthermore, studies in older professional athletes and spontaneously hypertensive rats have demonstrated that exercise increases SIRT3 and SOD2 expression, leading to an augmentation of tissue antioxidant capacity and attenuation of oxidative stress-induced cellular damage (105).

Molecular analyses indicate that exercise training upregulates the SIRT1/PGC-1α/PI3K/AKT signaling axis, in so doing promoting mitochondrial biogenesis and reducing oxidative stress in myocardial infarction (MI) models. In addition, the AMPK/SIRT1 signalling cascade, which plays a crucial role in lipid metabolism, is significantly activated in response to exercise, leading to an improvement in lipid phagocytosis and restoration of lipid droplet homeostasis in nonalcoholic fatty liver disease (NAFLD) (106). Exercise has been further implicated in cell survival pathways, predominantly in cardiac and renal tissues, where it modulates the SIRT1/p53 axis to impede apoptosis in cardiomyocytes and kidney cells, thereby preventing early-stage diabetic nephropathy and myocardial dysfunction. Also, endurance exercise has been shown to stimulate autophagy and mitophagy, leading to higher mitochondrial turnover and metabolic efficiency in the cortical region of young male mice.

Emerging evidence point to that exercise reduces cleaved poly (ADP-ribose) polymerase (PARP) levels, a key marker of DNA damage and apoptosis, in multiple pathological models, including aging, menopause, diabetic cardiomyopathy and doxorubicin-induced cardiotoxicity. This observation suggests that consistent physical activity may serve as a protective mechanism against excessive PARP-mediated NAD+ diminution and apoptotic signaling (107).

Exercise-induced modulation of oxidative stress markers has been established through upregulation of catalase and manganese superoxide dismutase (MnSOD) expression via the SIRT3/FOXO3a pathway, conferring cardioprotective effects in models of myocardial infarction. Furthermore, physical training has been shown to inhibit neuroinflammatory signaling cascades, particularly through downregulation of NF-κB and concurrent activation of the AMPK/SIRT1/PGC-1α axis, thereby reducing neuroinflammatory burden in diabetic and ApoE−/− mouse models (108).

Longitudinal studies additionally indicate that twenty weeks of structured endurance training significantly enhances SIRT3 protein expression in the hippocampus of amyloid precursor protein/presenilin-1 (APP/PS1) transgenic mice, a finding that correlates with attenuated neurodegenerative progression and enhanced cognitive function. These findings collectively underscore the role of exercise as a critical modulator of NAD+ metabolism, mitochondrial homeostasis and age-associated cellular deterioration, providing compelling evidence for its integration into clinical strategies aimed at mitigating ageing-related metabolic dysfunction (109).

## 5 Therapeutic implications and future directions

Exercise has been known as a key modulator of NAD+ metabolism, offering significant therapeutic benefits for overall metabolic health, mitochondrial function and cellular resilience. Numerous studies have demonstrated that regular physical activity efficiently enhances NAD+ levels, supporting the complex cellular processes that depend on this essential coenzyme (110, 111).

For instance, Brandauer et al. (112) found that a 3-week exercise regimen consisting of 1–2 h per session at 70%–85% peak workload significantly increased NAD+ levels and improved mitochondrial efficiency. Similarly, a study reported that 3 weeks of exercise, either 30–60 min at 75%–85% VO₂max or 50 min at 70% VO₂max per session, resulted in enhancements in oxidative capacity and NAD+ turnover, indicating that even relatively short-term exercise interventions can enhance NAD+ metabolism (113).

Evidence suggested that younger participants who engaged in 8 weeks of aerobic exercise at 65% VO₂max exhibited enhanced NAD+ biosynthesis and improved mitochondrial function. These findings underscore the ability of exercise to support NAD+ production and mitochondrial efficiency. In contrast, older participants showed less pronounced effects, suggesting that the ability to enhance NAD+ levels through exercise may vary across populations (114).

The potential of resistance training to modulate NAD+ metabolism was also highlighted by Lamb et al. (19), who observed significant increases in NAD+ levels and mitochondrial biogenesis after 10 weeks of resistance exercise, comprising 10–12 repetitions × 3 sets of whole-body exercises. These results demonstrate the therapeutic value of resistance training in improving NAD+ metabolism and overall cellular function.

Furthermore, long-term endurance exercise has been shown to sustain NAD+ production. Studies suggested have shown that regular endurance training (>1 h, 6 times per week) leads to sustained NAD+ production over time, with similar NAD+ levels observed in both young and older individuals. This suggests that consistent physical activity, especially endurance exercise, supports long-term NAD+ maintenance, enhancing cellular resilience and metabolic efficiency (115–117).

Collectively, these studies emphasize the therapeutic potential of exercise as a powerful strategy to boost NAD+ levels and improve mitochondrial function. Regular exercise, whether through aerobic, resistance, or endurance training, supports NAD+ metabolism, promotes cellular efficiency and enhances overall metabolic health. These findings have been supported by recent evidence showing that people who systematically participate in sports have significantly higher NAD+ levels in their erythrocytes than non-athletic controls, further highlighting the importance of long-term physical activity to maintain NAD+ homeostasis (12). Given its profound impact on NAD+ levels, exercise is an effective non-pharmacological approach that can support cellular health and metabolic function, benefiting individuals across various populations.

### 5.1 Exercise modality-specific effects on NAD+ metabolism

Various types of exercise have unique effects on NAD+ metabolism. AMPK–SIRT1–PGC-1α signalling pathway is consistently activated by endurance exercise, leading to mitochondrial biogenesis. High intensity interval training (HIIT) evokes high metabolic stress and short-lived boosts in NAD+ turnover. Sprint interval training (SIT) has shown mixed results, perhaps because of varying levels of fitness among participants and length of intervention. Resistance training primarily increases the expression of NAMPT, NAD+ levels in muscle and global sirtuin activity. This indicates that the amount and type of exercise-related adaptations in NAD+-related parameters could be modulated by an exercise prescription. Collectively, the available evidence suggests that endurance exercise and HIIT exert the most consistent effects on mitochondrial NAD+-related adaptations, whereas resistance training appears particularly beneficial for preserving skeletal muscle NAD+ metabolism during aging.

## 6 Conclusion

In conclusion, exercise plays a pivotal role in modulating NAD+ metabolism by upregulating the expression and activity of enzymes involved in NAD+ biosynthesis and consumption, such as NAMPT, NMNAT and sirtuins. Both aerobic and resistance exercises have been shown to increase NAD+ levels and improve mitochondrial function, thereby enhancing cellular resilience and mitigating the metabolic consequences of aging. These findings underscore the potential of exercise as an effective non-pharmacological intervention. Future study should aim to elucidate the tissue-specific effects of exercise on NAD+ metabolism, particularly in relation to exercise type, intensity and duration. A deeper understanding of these processes will inform the development of exercise-based therapeutic strategies to prevent or reverse age-associated metabolic decline.
